# An environmental scan of librarian involvement in systematic reviews at Queen’s University: 2020 update

**DOI:** 10.29173/jchla29517

**Published:** 2021-08-01

**Authors:** Amanda Ross-White

**Affiliations:** Health Sciences Librarian, Nursing Queen’s University, Kingston, ON

## Abstract

**Introduction:**

Systematic reviews are a growing research methodology in the health sciences, and in other disciplines, having a significant impact on librarian workload. In a follow up to an earlier study, an environmental scan was conducted at Queen’s University to determine what has changed, if anything, since the introduction of a tiered service for knowledge synthesis by examining review publications where at least one co-author was from Queen’s University.

**Methods:**

A search was conducted in PubMed and the Joanna Briggs database to find systematic reviews and meta-analyses with at least one author from Queen’s University for the five-year time since the last environmental scan. Reviews were categorized by the degree of involvement of the librarian(s) regardless of their institutional affiliation: librarian as co-author, librarian named in the acknowledgements, no known librarian involvement in the review.

**Results:**

Of 453 systematic reviews published in the five-year time frame, nearly 20% (89) had a librarian named as co-author. A further 24.5% (110) acknowledged the role of a librarian in the search, either in the acknowledgements section or in the body of the text of the article. In just over half of reviews (235 or 51.8%) a librarian was either not involved, or was not explicitly acknowledged. More librarians and more institutions were represented in the period of 2016-2020 than in 2010-2015.

**Conclusion:**

In the five years since the last environmental scan, an increasing number of reviews recognized the role of the librarian in publishing systematic reviews, either through co-authorship or named acknowledgement. This also suggests that as more librarians have become involved in systematic reviews, librarian capacity for this work has increased compared to five years ago.

## Introduction

Systematic reviews are a growing research methodology in the health sciences, as well as in other disciplines, which has had a significant impact on librarians’ workloads. For this reason, several librarians and library administrations have sought ways to manage both their workloads, as well as researcher expectations for what help librarians are able to provide [2-4]. In 2015, Ross-White conducted an environmental scan of systematic reviews published by researchers affiliated with Queen’s University to determine to what degree librarians were collaborating on these reviews. This prompted the development of a tiered service, which would allow librarians to choose their level of engagement with the project [5]. This paper seeks to determine what, if anything, has changed since the development of this service. Are librarians more, or less likely, to be included as co-authors? Do librarians receive named acknowledgment in systematic review publications for their role in conducting or consulting on the search?

In 2005, Sampson and McGowan advocated for librarian co-authorship on systematic reviews [6]. Major review publishers, including the Cochrane Collaboration and the Joanna Briggs Institute, have recommended consulting with a librarian for systematic review searching [7,8]. Since that time, multiple papers have explored the impact of this increasing role on librarians' workloads[2,9], looking at ways to increase capacity among librarians[10], time management of systematic review tasks[2,3], impact on burnout rates [11] and perceptions of the value of this work, particularly by senior management in health libraries.

Librarians are being asked to justify the use of their time on systematic reviews to senior library management, many of whom are not familiar with the methodology [14,15]. For this reason, it is important to know exactly how often librarians are involved in systematic review work, and to what degree they receive credit for this work. An increase in librarian involvement, either at the co-authorship level or at the acknowledgement level, is an indication that faculty and researchers who publish systematic reviews recognize and value the contributions of librarians.

## Methods

In order to replicate the previous environmental scan as closely as possible, we used the same search strategy, which was initially developed by Rethlefson and Montori[12,13]:

(search*[tiab] OR meta-analysis[Publication Type] OR meta-analysis[tiab] OR MEDLINE[tiab] OR (systematic[tiab] AND review[tiab]))]) OR systematic[sb] AND (”Queen’s University” [ad] OR ”Kingston General Hospital” [ad] OR “Kingston Health Sciences Centre ”[ad])

Only one small change was made to reflect the name change of our affiliated hospital from Kingston General Hospital to Kingston Health Sciences Centre, which occurred in 2017. As before, both PubMed and the JBI database were searched due to the affiliation of Queen’s University with the Joanna Briggs Institute. The search was conducted on November 13, 2020, and results were imported into EndNote software (version X9) for analysis.

Reviews were categorized based on the level of librarian involvement regardless of their institutional affiliation: librarian as co-author, librarian acknowledged (either by name or position in either the acknowledgements section or the body of the paper) and no known librarian involvement. Librarians were identified by position title or academic credentials, such as the Master of Library and Information Science, or similar degree.

## Results

From the search, 731 results were imported into EndNote. Of these, 456 articles were systematic reviews, with the remaining 275 being other types of references, such as commentaries or articles about systematic review methodology.

**Fig. 1:**
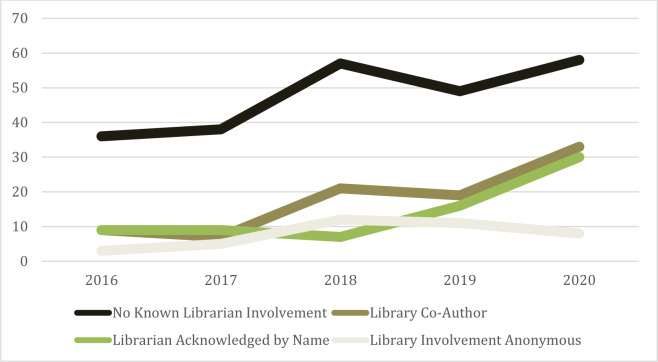
Authorship over the 2016-2020 period

Of the 456 systematic reviews published by at least one Queen’s University author, 89 listed a librarian as co-author, or nearly 20% (Table 1). A further 110 provided acknowledgement of the librarian’s role in the search, either in the acknowledgements or in the main body of the article. This made up 24.5% of the reviews. Of these, 71 provided the name of the librarian, and a further 39 only acknowledged the librarian by title or description within the body of the text. 238 articles either did not have a librarian involved, or did not explicitly provide credit to a librarian in some capacity. See Figure 1 for graphed authorship over the 2016-2020 period. The remaining 19 articles could not be obtained, so the librarian's role could not be determined.

**Table 1:** Involvement of Librarian(s) in reviews by Authorship / Acknowledgement

	No Known Librarian Involvement	Librarian Co-Author	Librarian Acknowledged by Name	Library Involvement Anonymous	Total
2016	36	9	9	3	57
2017	38	7	9	5	59
2018	57	21	7	12	97
2019	49	19	16	11	95
2020	58	33	30	8	129
	**238**	**89**	**71**	**39**	**437**

Acknowledgements followed a similar pattern to those in the previous study, with most librarians being identified by name. Examples include:

- “We gratefully acknowledge the assistance of Risa Shorr with developing the literature search strategy” [14].
- “The authors would like to thank the Holland Bloorview librarian, Pui Ying Wong, for her guidance and mentorship during the development of the search strategy” [15].
- “The authors would like to thank Sandra McKeown, Queen's University, for updating the literature search up to January 2019” [16].
- However, in some instances, the librarian remained anonymous, or only the library as a department was named.
- “A combination of alternate headings and key words were used following consultation with a scientific librarian” [17].
- “The authors thank the library staff at Mayo Clinic Arizona and the Countway Library of Medicine at the Harvard Medical School” [18].
- In one instance, the librarian was given initials, but no further information, making it impossible to determine who actually performed the search:
- “An experienced clinical librarian (TAW) conducted a literature search in the following electronic databases” [19].
- In another, neither the librarian who performed the search, nor the librarian who peer reviewed it, was named:
- “These terms were then used to perform a systematic search of the literature, which was conducted by a librarian. The search strategy was peer reviewed by a second librarian” [20].

## Discussion

When compared with the earlier environmental scan of systematic reviews at Queen’s University, it is clear that librarians are increasingly receiving both co-authorship and acknowledgement of their role in designing and executing searches in support of systematic reviews. Previously, 31/231 or 13.4% of systematic reviews had a librarian co-author. While 36/231 or 15.6% of systematic reviews had a librarian who received acknowledgement [21]. Combined with the total number of published reviews nearly doubling during the same period, it is understandable that librarians who perform systematic reviews would feel burnout[11].. Yet this number is still less than predicted by academic medical library managers who believe that the librarians reporting to them are acknowledged or listed as co-authors either ‘most of the time’ or ‘all of the time’ [13].

There is also a significantly greater number of librarians participating in reviews, in both academic and hospital libraries. Where the earlier scan showed most of the librarians were Queen’s University affiliated (22/31, or 71% in 2015), in the 2016-2020 period only 16 papers were co-authored by a librarian at Queen’s University, and 73 were co-authored by a librarian at another institution, representing just under 18% of the reviews considered for this study. In instances where a librarian received acknowledgement, the authors of 22 of systematic reviews recognized a librarian from Queen’s University, and 49 reviews acknowledged librarians from other institutions, representing 31% of the reviews considered for this study. This increase in external librarian co-authorship and acknowledgement is likely, in part, related to the uptake of systematic review (SR) responsibilities since 2015 by the profession. When the previous study was completed, fewer librarians, even those working in health sciences, were trained specifically in systematic review searches. The MLA SR project is just one example of how librarians have been formally trained in systematic review searching in the intervening years.

It is unclear what role the introduction of a tiered service may have played in the increase in librarians participating in knowledge syntheses or the increase in Queen’s librarians receiving acknowledgement and co-authorship. The tiered service likely precipitated a conversation around authorship and set expectations around what work could be expected from the librarian [5]. It is unknown how often the new systematic review form developed for the tiered service was used and how often librarians voluntarily chose not to pursue co-authorship, either due to workload issues or other concerns.

The range of institutions represented was wide. Hospital librarians and corporate, or special, librarians also received co-authorship in some instances. While a majority of acknowledgements and co-authorships were for other academic librarians across Canada (librarians from University of Toronto, University of Alberta, etc.), there were also hospital and specialist librarians (Holland Bloorview, Health Sciences Library at St. Michael’s Hospital, etc.), as well as international institutions represented (University of Michigan, University of Newcastle, University of Sydney). This is a notable increase since the previous study and shows that co-authorship is not only granted for academic librarians who may want or require publications for promotion or tenure, but it may also be perceived as a fair representation of the work involved. Hospital librarians do not receive immediate tangible benefits for authorship of papers in the same way academic librarians do, through the tenure or promotion system.

While the International Committee of Medical Journal Editors (ICMJE) has clear standards for authorship, it is not apparent from the reviews included in this study what the difference in workload might be between librarians who receive authorship, librarians who receive named acknowledgement and those who are unnamed. ICMJE requirements are that “contributors who have made substantive intellectual contributions to a paper are given credit as authors” which includes four areas of contribution:

- Substantial contributions to the conception or design of the work; or the acquisition, analysis, or interpretation of data for the work; AND
- Drafting the work or revising it critically for important intellectual content; AND
- Final approval of the version to be published; AND
- Agreement to be accountable for all aspects of the work in ensuring that questions related to the accuracy or integrity of any part of the work are appropriately investigated and resolved [22].

Those who meet any of the four criteria (but not all) should be acknowledged. The ICMJE also indicates that these criteria are not to be used as a means of exclusion, in that those who meet the first criteria of substantial contributions to the work must be provided an opportunity to meet the remaining criteria. As an example, authors cannot withhold a draft of the paper from someone who contributed to it substantially just to ensure they cannot approve of the final version, the third criteria in the ICMJE checklist for authors.

Research on time measurement of systematic review tasks has found that the search is the most substantial contributor to systematic review workload for librarians, and that the additional three tasks required for co-authorship are not likely to be onerous[2,23]. In all instances, being a named contributor through acknowledgement would appear to be a requirement given that the search consists of a “substantial contribution to the conception or design of the work”[22]. With systematic reviews that have librarians as members of the team consistently being of higher quality in many disciplines, the need for a librarian to be an active participant in the team is worth the time investment so the institution can improve its research profile [12,24,25].

### Limitations

In order to replicate the previous study as closely as possible, we did not make changes to our search to reflect a move to different types of knowledge syntheses, such as scoping reviews [26]. In addition, the search was limited to two databases, PubMed and the Joanna Briggs Collaboration, meaning the growth of systematic reviews in other disciplines such as psychology, engineering or education may be underrepresented. As a result, some reviews published by Queen’s University authors were not captured by this search.

## Conclusion

Over the past five years, the role for librarians in systematic reviews has increased, along with both the overall numbers of reviews being published and the level of responsibility involved. This environmental scan does not account for unpublished reviews that were supported by librarians. It is apparent from the results that the capacity for reviews has also increased, with an increasing number of librarians in academic, hospital and corporate settings supporting systematic reviews enough to receive co-authorship or named acknowledgement. Librarians should continue to advocate for the appropriate level of acknowledgement given their role so that credit can be given where it is due.
